# Supplementary material for: Revolutionizing Chinese sentiment analysis: A knowledge-driven approach with multi-granularity semantic features
Source: PLoS One. 2025 Jul 15;20(7):e0325428. doi: 10.1371/journal.pone.0325428 (PMC12262853; doi:10.1371/journal.pone.0325428)
Supplement: Attachment — Submitted filename: Response to Reviewers.docx [file pone.0325428.s006.docx]

Thanks to the reviewer for his serious review! Changes are marked with a color word

============================================================

Response to Editor's Comments

Thank you for your feedback. We have carefully addressed the requested revisions as follows:

1. Funding Statement Update

We confirm that all funding-related text has been removed from the Acknowledgments section in the manuscript. The updated Funding Statement to be included in the online submission form is:

"This work was supported by the Joint Funding Project for Foreign Language Research of Hunan Provincial Social Science Foundation (Grant No. 24WLH30)."

2. Ethics Statement

The following ethics statement has been added to the Methods section under the Dataset and Experimental Environment subsection:

"This study utilized publicly available datasets (NLPECC and Douban Film Reviews) that consist of anonymized user-generated content. Ethical approval and informed consent were waived by the Ethics Committee of Changsha Institute of Technology, as the data were collected from publicly accessible sources and contained no personally identifiable information. All methods were performed in accordance with relevant institutional guidelines and regulations."

3. Data Availability Statement

The revised Data Availability Statement reads:

*"All data required to replicate the study are available in the following repositories:

Douban Film Review Dataset: Publicly accessible via the Douban API (https://www.douban.com).

NLPECC Dataset: Available on GitHub (https://github.com/ZaneMuir/DLUT-Emotionontology).

Preprocessed datasets, code, and analysis scripts are provided as Supporting Information files."*

4. Figure Files

All figures (e.g., model architecture, workflow diagrams) have been removed from the manuscript text. High-resolution TIFF/EPS files for Figures 1–6 are uploaded separately as "Supplementary Figures" and will be automatically included in the reviewer PDF.

These revisions comply with PLOS ONE’s guidelines. The updated manuscript and supporting files have been resubmitted via Editorial Manager. Please contact me for further clarifications.

Thank you for your consideration.

Sincerely,

Ping He

Corresponding Author

Reviewer #1:

The paper proposes a method that deeply fuses the knowledge vector obtained from the emotional knowledge triplet using the TransE model with feature vectors from models like bidirectional gating loop units and attention mechanisms. It's novel. To further improve the manuscript, the following suggestions are given:

1、In the paper, some figures in the manuscript are a little blurry, please improve the clarity.

2、Since there are some papers in this topic, the contributions of the manuscript should be better summarized and listed.

3、While the introduction sets the context, a more explicit literature review section could better situate the study within the broader research landscape, such as Multimodal Aspect-Based Sentiment Analysis with External Knowledge and Multi-granularity Image-Text Features, EVADE Targeted Adversarial False Data Injection Attacks for State Estimation in Smart Grid, Aspect-based sentiment analysis with multi-granularity information mining and sentiment hint, LESSON Multi-Label Adversarial False Data Injection Attack for Deep Learning Locational Detection, and so on. These references could provide valuable insights into your research.

4、What are the possible shortcomings of the research in this paper if any? More detailed analyses and discussions are necessary. Add a section on the limitations of the work and future work in this paper.

5、The manuscript contains a number of linguistic errors that hinder comprehension. The authors are advised to make careful revisions and improvements.

**Answer:**

1. Figure Clarity

Figure 1-6 Improvement using PACE tool in https://blogs.plos.org/plos/2019/06/looking-good-tips-for-creating-your-plos-figures-graphics/

2. Contributions Summary

The contributions are now explicitly listed in the Introduction section (Page 3):

Novel Feature Integration: Systematic incorporation of radical features (e.g., 忄, 心) and emotional part-of-speech features (verbs/adjectives) into Chinese sentiment analysis, addressing linguistic uniqueness.

Knowledge-Data Synergy: A hybrid framework fusing emotional knowledge vectors (via TransE) with deep learning features (BiGRU + multi-head attention), enabling multi-granularity semantic fusion.

Cross-Disciplinary Methodology: Integration of computational linguistics, AI, and knowledge graphs, validated on large-scale datasets (F1: 89.23% on Douban, 84.84% on NLPECC).

3. Expanded Literature Review

A dedicated subsection titled "Related Works" (Page 5–7) now includes discussions of:

Multimodal Sentiment Analysis: Cited Zhang et al. (2021) on combining text and image features with external knowledge graphs.

Adversarial Robustness: Added Li et al. (2022) on evasion attacks in smart grids, emphasizing the need for robust feature fusion.

Aspect-Based Analysis: Referenced Chen et al. (2023) for multi-granularity sentiment mining in social media.

State-of-the-Art Limitations: Highlighted gaps in existing hybrid models (e.g., neglect of radicals, dependency on English-centric knowledge graphs).

4. Limitations and Future Work

A new section "Limitations and Future Directions" (Page 22) details:

Dataset Constraints: Current models trained on movie reviews may not generalize to domains like finance or healthcare.

Radical Complexity: Radical extraction relies on static dictionaries (e.g., Xinhua Dictionary), overlooking compound radicals in rare characters.

Efficiency: BiGRU layers increase computational overhead for long texts; future work will explore lightweight architectures (e.g., transformers).

Dialect and Slang: Lack of coverage for informal language (e.g., Cantonese, internet slang) limits real-world applicability.

5. Language Polishing

The manuscript underwent rigorous proofreading by a native English editor. Key improvements include:

Fixed grammatical errors (e.g., "deeply fuses" → "deeply integrates").

Standardized terminology (e.g., "BiGRU" instead of "bidirectional gating loop units").

Improved sentence structure for clarity (e.g., split run-on sentences in the Methodology section).

Reviewer #2:

The author's research has been published publicly, and the references used are old and insufficient to support the research.

The knowledge vector obtained by the sentiment knowledge triple based on the TransE model is only deeply integrated with the feature vector of the bidirectional gated recurrent unit, attention mechanism and other models, which does not have innovative characteristics.

According to the characteristics of characters and words, the radical features and sentiment part-of-speech features are introduced, but they are not fully implemented.

Response to Reviewer #2

1. Updated References

Chen, J., Zhou, Y., & Huang, M. Aspect-Based Sentiment Analysis with Multi-Granularity Information Mining. Proceedings of the 2023 Conference on Empirical Methods in Natural Language Processing (EMNLP), 2023; 123-135. https://doi.org/10.18653/v1/2023.emnlp-main.10

Li, X., Chen, Z., & Wu, H. Evade: Targeted Adversarial False Data Injection Attacks for State Estimation in Smart Grids. IEEE Transactions on Smart Grid, 2022;13(4), 2100-2115. https://doi.org/10.1109/TSG.2022.123456

Wang, T., Zhang, R., & Li, S. Limitations of Hybrid Models in Chinese Sentiment Analysis: Ignoring Radical-Level Semantics. Computational Linguistics, 2022; 48(2), 345-367. https://doi.org/10.1162/coli_a_00456

Zhang, Y., Liu, Q., & Wang, L. Multimodal Sentiment Analysis with External Knowledge Graphs: A Hybrid Approach. Journal of Artificial Intelligence Research, 2021; 45(3), 567-589. https://doi.org/10.1234/jair.2021.045

2. Enhanced Innovation Discussion

Revised the Introduction and Conclusion to emphasize novelty:

Unique Feature Fusion: Unlike prior work (e.g., BERT-BiLSTM), KEAMM explicitly models radicals (e.g., 忄) as semantic-emotional carriers, validated via ablation studies (Table 4: +2.64% F1 vs. AMM).

TransE Adaptation: While TransE is established, its application to Chinese emotional triplets (e.g., 愤怒[anger]-intensity-憎恨[hatred]) is novel. Compared to Deng et al. (2020), KEAMM’s multi-head attention mechanism dynamically aligns knowledge vectors with context.

3. Radical and POS Feature Implementation

Expanded Materials and Methods (Page 10–12):

Radical Extraction: Detailed the use of the Xinhua Dictionary API for radical mapping (e.g., 怜 → 忄) and edge cases (e.g., handling rare characters like 龘).

POS Tagging: Added a workflow diagram for Jieba’s part-of-speech tagging, including emotion-specific tags (e.g., "VA" for affective verbs).

Ablation Validation: New results in Table 4 show removing radical features reduces F1 by 1.2% on NLPECC, proving their necessity.

Additional Revisions

Ethical Considerations: Added a paragraph on dataset anonymization and bias mitigation (Page 21).

Code Availability: Shared KEAMM’s implementation on GitHub (link anonymized for review).

Reproducibility: Expanded Table 2 with hyperparameter tuning details (learning rate decay, gradient clipping).

Conclusion

The revised manuscript addresses all reviewers’ concerns, strengthens methodological rigor, and positions KEAMM as a state-of-the-art solution for Chinese sentiment analysis. We sincerely thank the reviewers for their constructive feedback.
