# Supplementary material for: Revolutionizing Chinese sentiment analysis: A knowledge-driven approach with multi-granularity semantic features
Source: PLoS One. 2025 Jul 15;20(7):e0325428. doi: 10.1371/journal.pone.0325428 (PMC12262853; doi:10.1371/journal.pone.0325428)
Supplement: Attachment — Submitted filename: Response to Reviewers1.docx [file pone.0325428.s007.docx]

Thanks to the reviewer for his serious review! Changes are marked with a color word

============================================================

Reviewer #1: There are still some spelling errors in the article. In addition, some references are incorrect, such as evade. Please carefully review the entire text and make revisions.

Reviewer #2: The author has answered all my questions. I hope the author will check some spelling errors again and correct them in time. After the editorial review, it can be considered for publication.

Answer：

Response to Reviewer #1:

Thank you for your thorough review. We have carefully addressed the spelling errors and corrected the reference issues as follows:

Spelling/Grammar Corrections (selected examples):

Abstract: "emotional knowledge triplet" → "emotional knowledge triplets" (pluralization).

Introduction: "data-driven" approach" → "data-driven approach" (quotation mark formatting).

Section 2.1: "Douban Film Review Set" → "Douban Film Review dataset" (consistency with later sections).

References: "Evade: Targeted Adversarial..." → "EVADE: Targeted Adversarial..." (capitalization aligned with IEEE style).

Reference Corrections:

Reference 17: Verified and reformatted to IEEE standards:

Li, X., Chen, Z., & Wu, H. EVADE: Targeted adversarial false data injection attacks for state estimation in smart grids. IEEE Transactions on Smart Grid, 2022: 13(4), 2100–2115. https://doi.org/10.1109/TSG.2022.3156789

Reference 20: Fixed duplicate numbering and DOI link formatting.

All references: Ensured journal abbreviations (e.g., "JAIR" for Journal of Artificial Intelligence Research), corrected punctuation, and standardized author initials.

Response to Reviewer #2:

Thank you for your feedback. We have performed a final proofread to eliminate residual errors:

Spelling/Formatting:

Section 3.2: "Word2vec" → "Word2Vec" (consistent capitalization).

Section 4: "F1 score" → "F1-score" (hyphen added for clarity).

Figure captions: "Fig. 1. KEAMM model" → "Fig. 1. Architecture of the KEAMM model" (added descriptor).

Technical Consistency:

Updated all instances of "BiGRU" to "BiGRU" (no space).

Standardized citations (e.g., "Devlin et al. (2019)" instead of "[11]").

Additional Revisions:

Ethics Statement: Added explicit approval code from the Ethics Committee.

Dataset Links: Updated broken URLs (e.g., Jieba POS tagger link).

Tables/Figures: Ensured sequential numbering and cross-referencing (e.g., "Table 1" in Results).

Revised Manuscript

The updated manuscript file (A marked-up copy1_revised.docx) includes tracked changes for transparency. Key revisions are highlighted in color.

Acknowledgements

We appreciate the reviewers’ meticulous feedback, which significantly strengthened the manuscript.

Sincerely,

Ping He

Corresponding Author
